# Development and testing of Schisto and Ladders™, an innovative health educational game for control of schistosomiasis in schoolchildren

**DOI:** 10.1186/s13104-017-2545-5

**Published:** 2017-06-28

**Authors:** Cynthia Uchechukwu Ejike, Akinola Stephen Oluwole, Hammed Oladeji Mogaji, Adebiyi Abdulhakeem Adeniran, Oladimeji Michael Alabi, Uwem Friday Ekpo

**Affiliations:** Spatial Parasitology and Health GIS Group, Department of Pure and Applied Zoology, Federal University of Agriculture, Abeokuta, Nigeria

**Keywords:** Schistosomiasis control, Health education game, Schisto and Ladders™ knowledge, Attitude, Practices, Schoolchildren, Prevention

## Abstract

**Background:**

Schistosomiasis remains a public health problem in many regions of the world, including Nigeria. Current control strategy involves mass drug administration with praziquantel to the endemic population. To complement and sustain on-going preventive chemotherapy, we developed a health educational game named Schisto and Ladders™ and tested its potential for the control of schistosomiasis among schoolchildren living in Imala-Odo, a highly endemic community near Abeokuta, Nigeria.

**Methods:**

One hundred school children were randomly selected and divided into intervention and control groups through balloting. Their knowledge, attitudes and practices (KAP) concerning schistosomiasis transmission, control and prevention were assessed using structured questionnaires. Schisto and Ladders™ game were given to the intervention group and the popular Snake and Ladders™ game to the control group. Both games were played for 2 months under the supervision of their class teachers. A post-KAP assessment was carried out in both groups, including focus group discussions (FGDs) to investigate knowledge and the impact of the games.

**Results:**

Knowledge about urinary schistosomiasis and its transmission significantly improved (P = 0.000) in the intervention group (68.0%) compared to the control group (8.0%). FGDs showed that the frequency of visits to dam water also significantly reduced (P = 0.048) in the intervention group (18.0%) compared to the control group (40.0%). There was a significant increase in knowledge regarding risk behaviours, prevention and control of schistosomiasis among the intervention group, but no new knowledge gained in the control group.

**Conclusions:**

This study demonstrates the potential of the health education game Schisto and Ladders™ for teaching basic health education and promoting behavioural changes among schoolchildren in endemic communities.

## Background

Schistosomiasis remains a public health challenge with over 600 million people at risk of infection worldwide [1–3]. The transmission of the disease is linked to contact with infected water bodies such as rivers, streams, pools and lakes containing cercaria in endemic communities [4]. Schistosomiasis is common in school-aged children whose water contact pattern includes recreational and domestic activities such as bathing, swimming, washing clothes or kitchen utensils and fetching of water, which are common among primary school children between the ages of 5–15 years old [5–8].

Current control of schistosomiasis is majorly through preventive chemotherapy treatment with Praziquantel either school-based or community-based [4]. Although preventive chemotherapy substantially reduces the infection burden and parasite-associated morbidity, immediate re-infection is not usually prevented, and school children in high-risk communities get re-infected rapidly requiring repeated treatment [9, 10]. Recent studies in Mali and Kenya have shown the persistent prevalence of schistosomiasis in school children after repeated preventive chemotherapy [11, 12]. However, evidence exists suggesting that proper school-based health education can influence health-seeking behaviour and reduce prevalence of infection among school children [13]. Hewlett and Cline [14] adapted health education to local conditions in Cameroon, targeting health education at children. Their study reported a dropped in prevalence from 21 to 7% after 2 years. Complimenting health education with deworming program has been suggested as a potential way of breaking parasite’s transmission cycle by preventing contamination of the environment [15]. Complementary interventions such as improvement in water and sanitation [16] and health education have been advocated, as additional efforts essential to sustain the gains of preventive chemotherapy [17, 18]. It is against this background we are reporting a newly developed health educational game Schisto and Ladders™ and its potential to promote behavioural changes for schistosomiasis control by increasing knowledge of schistosomiasis among school children in an endemic community near Abeokuta, Nigeria.

## Methods

### Study design

The study was an intervention trial involving an intervention group that played the Schisto and Ladders™ game and a control group that played the Snake and Ladders (placebo) for 2 months. Quantitative and qualitative data were collected from both study groups. Pre-and post-test questionnaires administered to both groups were used to obtain quantitative data while qualitative data was collected during focus group discussions (FGD). Data were collected from April to August 2014.

### Study area

The study was conducted at Abeokuta North Local Government Primary School, Imala-Odo. The school is the only primary school in this community and has no toilet facility or water supply apart from the non-functional borehole sited in front of the school. Imala-Odo is a highly endemic community for schistosomiasis since 1991 after the establishment of the Oyan Dam [19]. This disease has persisted over the years in this community [20, 21]. The major occupation of members of the community is fishing and farming. There is no communal toilet facility, electric power supply, pipe borne water, good road network and proper waste disposal system. The community depends on the dam water for their domestic, recreational and occupational activities. An assessment of the prevalence of urogenital schistosomiasis in the school was 89.0%.

### Ethics considerations

Prior to commencement of study, meetings were held with the parents, caregivers, teachers and headmistress of the school children and were briefed about the objectives of the study. The community has previously been surveyed for schistosomiasis and was thus familiar with researchers which made the acceptance of the study easier [20]. Selected participants were given consent form from school which was taken home to be assented to by signing or thumb printing by parents. Ethical approval was granted by the ethical review board of Ogun State Ministry of Health, Abeokuta. Permission to enter the school was obtained from the State Universal Primary Education Board, Abeokuta.

### The Schisto and Ladders™ game

**Fig. 1:**
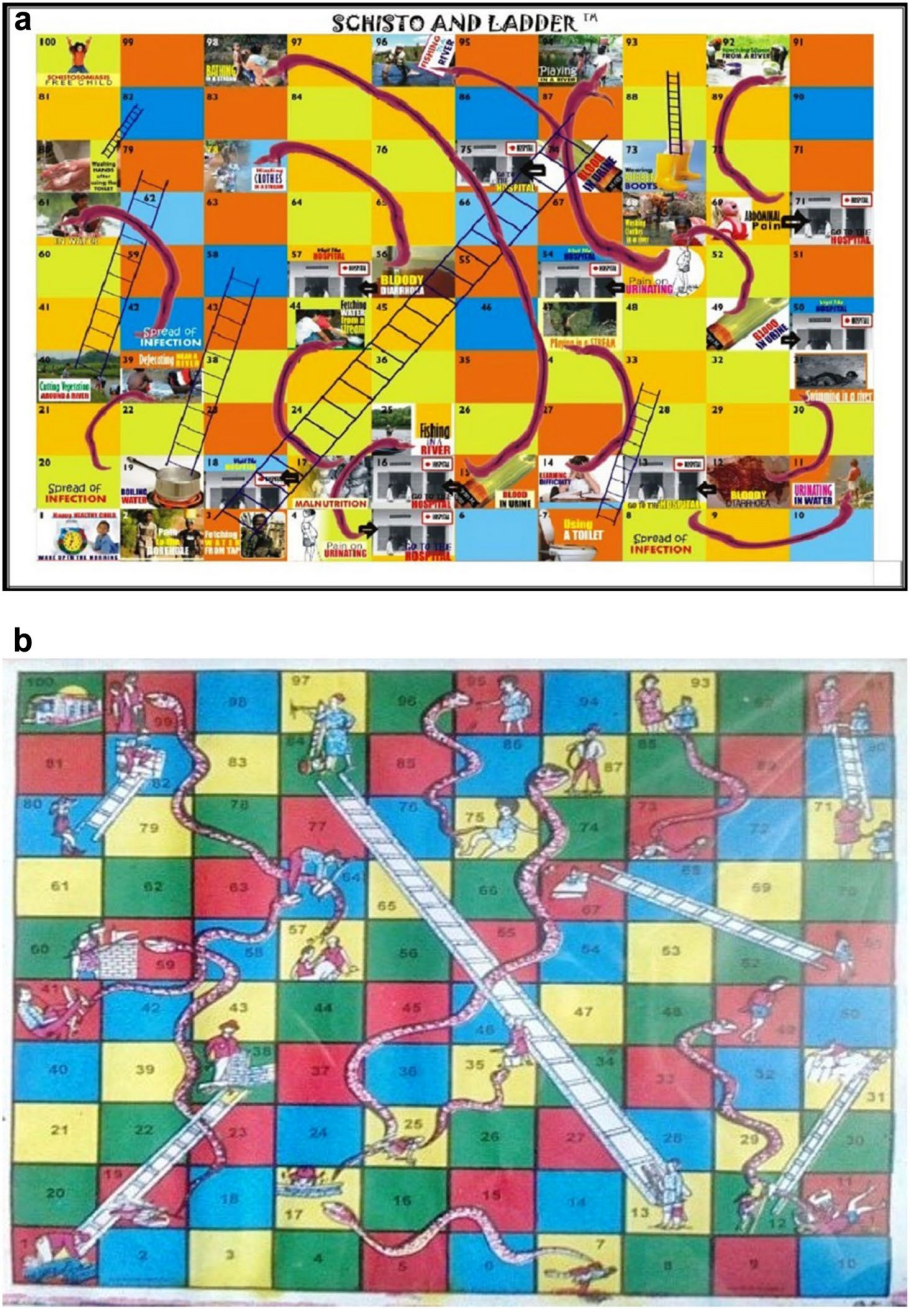
**a** The designed *Schisto and Ladders™* board game (test). **b** The common snake and ladder board game (control)

Schisto and Ladders™ game is a board game (Fig. 1a) designed from the popular Snake and Ladders game (Fig. 1b). The game aims to elicit positive behavioural changes in players by informing and educating the players about schistosomiasis, its transmission, control, and prevention. The game is based on the concept of reward for good health behaviours by moving up a ladder and punishment for risky health behaviours by being bitten by the *Schistosoma* worm. The game has several health education messages presented in the children-friendly pictorial form. Health educational messages presented in the board game include mode of transmission of the infective stage (cercaria), behavioural risks associated with transmission (risk factors for transmission of infection), symptoms of infection and information on what to do to seek treatment, prevention of reinfection and control strategies. The game can be played by a minimum of 2 and a maximum of 4 persons, to allow for proper interaction and assimilation of the health education messages and warnings on the board. The game starts when a player throws a 1 with a dice. A player moves up when he/she gets to the foot of a ladder and goes down when he gets to the head of the *Schistosoma* worm. Players are self-guided by the health messages on each square of the board. The players count the number shown on the dice when thrown and the first player to get to the square marked 100 wins the game.

### Testing of the Schisto and Ladders™ game

#### Questionnaire administration

**Fig. 2:**
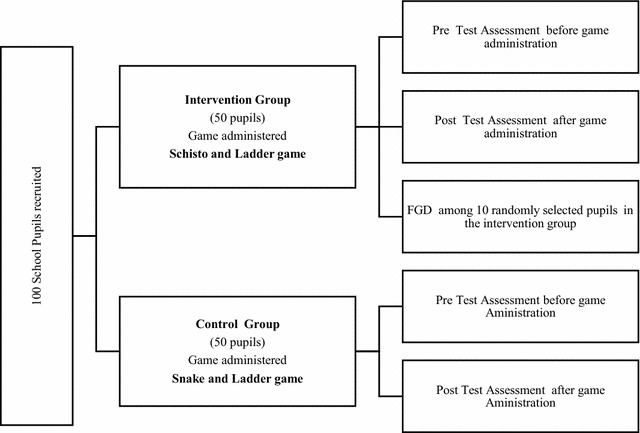
Flowchart for study design and selection of pupil for playing the test and control games

One hundred pupils were selected randomly through balloting from the school population. They were randomly split into two equal groups of 50 pupils for intervention and 50 pupils for control respectively (Fig. 2). A pre-test questionnaire specific to understanding participants’ knowledge, attitudes and practices (KAP) about schistosomiasis, its transmission, pathology and control were administered to both study groups. The health education board game Schisto and Ladders™ was distributed to the intervention group, and the ordinary Snake and Ladders board game were given to a control group. The pupils were taught how to play both games. The games were played daily among pupils of the same cadre during the 30 min long break for 2 months under the supervision of their class teachers. The person that came last in a round was replaced by someone else in the next set of the game. A post-test questionnaire was administered at the end of 2 months to both intervention and control groups. Participants in both intervention and control group including the teachers were blinded to the study objectives to eliminate bias.

### Focus group discussions

Focus group discussions (FGDs) on perception and knowledge gained from the games were held in the school among the study participants after the trials. The service of a trained social scientist was employed for the FGD. Ten pupils from each group without gender bias were selected randomly for the FGD. Sessions were held separately for the test and control groups. Some of the questions asked during the FGD include what activity makes one to be bitten by the Schisto worm to test for knowledge about risk behaviours, what are the symptoms of infection and what should be done when one discovered he/she is having symptoms of infection. A portable electronic voice recorder was used in recording the conversations of the FGDs. Qualitative data from the FGDs were coded on knowledge, attitude and practice change and was equally the basis on which the flow of the FGD was directed.

### Data analysis

Data obtained from questionnaires were entered into Microsoft Excel (Microsoft Corporation) and analysed using SPSS 16.0 software. Descriptive statistics that include frequency and percentages were used to describe data collected. Chi square was used to test for significant difference between quantitative variables. Qualitative data from FGDs were transcribed and analysed manually.

## Results

The ages of 100 pupils that participated in testing of the game ranged between 5 and 19 years (mean age = 11 years). 58% were females, and 42 (42.0%) were males. There were no significant difference in gender the intervention and control categories (P > 0.05). Table 1 shows the demographic characteristics of the study group.

**Table 1:** Demographic characteristic of study participants

Variables	Intervention group	Control group	Total (%)
	No. examined (%)	No. examined (%)	
Sex			
Male	22 (44.0)	20 (40.0)	42 (42.0)
Female	28 (56.0)	30 (60.0)	58 (58.0)
Total	50 (100.0)	50 (100.0)	100 (100)
P value = 0.854			
Age group (year)			
5–9	19 (38.0)	15 (30.0)	34 (34.0)
10–14	23 (46.0)	30 (60.0)	53 (53.0)
15–19	8 (16.0)	5 (10.0)	13 (13.0)
Total	50 (100.0)	50 (100.0)	100 (100)
P value = 0.352			
Parents’ occupation			
Farming only	11 (22.0)	10 (20.0)	21 (21.0)
Fishing only	4 (8.0)	5 (10.0)	9 (9.0)
Farming and fishing	33 (66.0)	33 (66.0)	66 (66.0)
Fishing and trading	–	2 (4.0)	2 (2.0)
Farming and trading	1 (2.0)	–	1 (1.0)
Fishing and tailoring	1 (2.0)	–	1 (1.0)
Total	50 (100.0)	50 (100.0)	100 (100)
P value = 0.527			

### The impact of Schisto and Ladders™ health education game on knowledge, attitude, and practices

#### Knowledge about disease and its symptom

Before the introduction of the game in the intervention group, only 14 (28.0%) pupils knew that blood in urine is a symptom of urogenital schistosomiasis, however, after playing the games for 2 months, knowledge about blood in urine as a symptom of schistosomiasis infection increased to 37 (74.0%). In the control group, 16 (32.0%) pupils believed that blood in the urine was a sign of disease before administration of the game and after game administration, 29 (58.0%) of the pupils responded that blood in urine is a disease condition. However, there was no significant difference in knowledge gained by both groups before (P = 0.828) and after (P = 0.139) intervention (Table 2).

**Table 2:** Knowledge about schistosomiasis among intervention and control group pre-and post-game administration

	Intervention group		Control group		P value
	Before intervention (%)	After intervention (%)	Before intervention (%)	After intervention (%)	
Blood in urine					
Yes	14 (28.0)	37 (74.0)	16 (32.0)	29 (58.0)	*0.828, **0.199
No	36 (72.0)	13 (26.0)	34 (68.0)	21 (42.0)	
Total	50 (100)	50 (100)	50 (100)	50 (100)	
Cause of blood in urine					
Schistosomiasis	0 (0)	34 (68.0)	0 (0)	4 (8.0)	*0.749, **<0.001
Stomach pain	5 (10.0)	2 (4.0)	6 (12.0)	5 (10.0)	
No Idea	45 (90.0)	14 (28.0)	44 (88.0)	40 (80.0)	
Snake	0 (0)	0 (0)	0 (0)	1 (2.0)	
Total	50 (100)	50 (100)	50 (100)	50 (100)	
Means of acquiring *Schistosoma* infection					
Contact with water in dam	1 (2.0)	45 (90.0)	3 (6.0)	22 (44.0)	*0.593, **<0.001
Stomach pain	2 (4.0)	0 (0)	2 (4.0)	0 (0.0)	
No idea	47 (94.0)	5 (10.0)	45 (90.0)	28 (56.0)	
Total	50 (100)	50 (100)	50 (100)	50 (100)	

* P value before intervention

** P value after intervention

Prior to administration of the game, 90% (45) of the pupils in the intervention group had no idea about the infection causing blood in urine while 5 (10.0%) perceived stomach pain as reason for having blood in urine. However, after the game administration (post-test), 34 (68.0%) correctly responded that schistosomiasis is the infection that causes blood in urine; 14 (28.0%) had no idea, and 2 (4.0%) perceived stomach pain as the infection. In the control group, 44 (88.0%) pupils did not know, while 6 (12.0%) perceived stomach pain as the infection that causes blood in urine before game administration. After game administration (post-test) 40 (80.0%); 1 (2.0) and 5 (10.0%) pupils had no idea, believe snake bite, and stomach pain respectively are the reasons for blood in urine. There was no significant difference between knowledge gained in both groups before (P = 0.749) game administration (pre-test) while there was a significant difference (P = 0.000) after game administration (Table 2).

#### Knowledge about source of infection

Knowledge about the source of infection before game administration in the intervention group revealed 47 (94.0%) pupils with no idea about where the infection can be acquired; other responses were 2 (4.0%) for stomach pain and 1 (2.0%) for bathing in the dam water. However, after game intervention (post- test), 23 (46.0%) perceived water contact activities as a risk behaviour of getting infected (Table 2). In the control, 45 (90.0%) had no idea about risk behaviours, before the game intervention. After game administration (post-test), 28 (56.0%) had no idea, 17 (34.0%) perceived all water contact activities relating to dam water as a risk behaviour. While other responses were 2 (4.0%) for bathing, washing, and fetching, 1 (2.0%) for bathing and fetching, 1 (2.0%) for Fetching, washing, bathing and fishing and 1 (2.0%) for bathing and washing (Table 2). There was no significant difference in knowledge in both groups before game administration (pre-test) (P = 0.711). However, after game administration (post-test), there was a significant difference (P = 0.000).

#### Water contact practices

Before game administration majority 29 (58.0%) of the pupils in the intervention group reported going to the dam water more than thrice in a day, while 19 (38.0%) of them visited thrice daily, and 2 (4.0%) of them visited twice daily (Table 3). However, after game administration (post-test), only 9 (18.0%) of the pupils visits the dam water more than thrice daily, 37 (74.0%) went thrice and 4 (8.0%) went twice daily (Table 3). In the control group, 32 (64.0) of the pupils visits the dam water more than thrice in a day, while 13 (26.0%) visits thrice and 5 (10.0%) visits twice before game administration. After game intervention (post-test), 26 (52.0%) had gone to the dam water twice daily, 20 (40.0%) went to the dam water more than thrice daily and 4 (8.0%) for twice in a day. There was no significant difference in daily frequency to the dam water between both groups before game administration (pre-test) (P = 0.278) while the difference observed was statistically significant after game administration (post-test) (0.048) (Table 3).

**Table 3:** Pre-and post- assessment of daily frequency of exposure to dam water

	Intervention group		Control group		P value
	Pre-test assessment (%)	Post-test assessment (%)	Pre-test assessment (%)	Post-test assessment (%)	
Daily frequency of exposure to dam water					
Twice	2 (4.0)	4 (8.0)	5 (10.0)	4 (8.0)	*0.278, **0.048
Thrice	19 (38.0)	37 (74.0)	13 (26.0)	26 (52.0)	
More than thrice	29 (58.0)	9 (18.0)	32 (64.0)	20 (40.0)	
Total	50 (100)	50 (100)	50 (100)	50 (100)	
Frequency of playing activities in dam water					
Not always	3 (6.0)	40 (80.0)	3 (6.0)	46 (92.0)	*0.761, **0.153
Always	42 (84.0)	8 (16.0)	44 (88.0)	4 (8.0)	
Never	5 (10.0)	2 (4.0)	3 (6.0)	0 (0)	
Total	50 (100)	50 (100)	50 (100)	50 (100)	

* P value before intervention

** P value after intervention (Chi squares were from 2 × 2 table with pre-and post assessment data analysed separately)

The pre-test assessment of the frequency of playing in the dam water in the intervention group revealed that 42 (84.0%) play in the dam water always, 3 (9.0%) seldom plays in the dam water, and only 5 (10.0%) of the pupils had never played in the dam water. However, after game administration (post-test), the number of pupils playing in the dam water always reduced to 8 (16.0%). While 40 (80.0%), not playing in the dam water always, while 2 (4.0%) had never played in the dam water (Table 3). The pre-test assessment in the control group, however, revealed that 44 (88.0%) pupils play in the dam water always, 3 (6.0%) does not play in the dam water always, and 3 (6.0%) had never played in the dam water. After game administration (post-test) 46 (92.0%) do not play the dam water always, and 4 (8.0%) play in the dam water always. There was no significant difference in the effects of the games on the frequency of playing in the dam water before administration (P = 0.761) and after administration (P = 0.153) (Table 3).

The result of FGD to assess the impact of the games administered in intervention and control groups is as shown in Table 4.

**Table 4:** FGD to assess the impact of Schisto and Ladders™ on KAP of intervention and control group

		Question asked to kick start discussion during FGDs	Intervention group	Control group
	Means of transmission of *S. haematobium*	What activities can make *Schistosoma* bite a player in Schisto and Ladders™ game?	Nine out of the 10 pupils involved in the FGD mentioned at least one of playing, fishing, bathing, fetching water and washing clothes in dam water as activity that will make *Schistosoma* bite a player	Only 4 pupils from the group could correctly state an activity related to *Schistosoma* bite with one boy that said all of fetching, washing and fishing in dam water which he said he learnt from his sister in the intervention group
Knowledge	How to avoid getting infected with *S. haematobium*	What activities promote access up the ladder in Schisto and Ladders™ game	Most of the pupils mentioned activities such as fetching water from a tap and using a toilet as activities that will make one go up the ladder	No pupil in the control group could give a correct response
	Symptoms of *S. haematobium infection*	What are the symptoms that show schistosomiasis infection	All the pupils could relate schistosomiasis infection with the presence of blood in urine, which they referred to as blood in piss and blood in shit as seen in the game played	No pupil in the control group gave a correct response
	Intermediate host for *S. haematobium* worm	What animal is associated with schistosomiasis	The majority of the children could link Schistosomiasis infection to water snails that were seen by the dam water banks in the Schisto and Ladders™ game while two of them linked it to goat and dog	None of the pupils could link, snail with schistosomiasis infection
Attitude	The most appropriate place to defecate	Where is it appropriate to defecate	All the pupils responded that a toilet should be used for defecating	There was no response from the pupils in this group
Practice	Measure to be taken when blood is seen in the urine	What would you do when blood is observed in the urine	All the pupils responded that when blood is seen in the urine, the sufferer should go to the hospital	None of the pupils had an idea about what to do when blood is seen in the urine
Perception	Acceptability of the administered game	Participants were asked during the FGDs on their perception of the game they played	When a suggestion was made by the facilitator for a swap of Schisto and Ladders™ game with Snake and Ladder game, the pupils in the intervention group disagreed with a loud NO. A girl said it (snake and ladder) does not have words in it. They preferred Schisto and Ladders™ because it had words written on it which helped them in learning	The pupils in control group all wanted a swap of the Snake and Ladder game with the Schisto and Ladders™ game because it does not have a word in it except a boy who could not give a reason while he preferred the Snake and Ladder game. Those in the control group complained that the Snake and Ladder game had nothing written on it, so they did not learn anything from it

## Discussion

It is now widely believed that to sustain the gains of mass drug administration for the control of schistosomiasis; there is the need for complementary intervention such as improvement in water and sanitation and health education [11, 16]. This study designed and tested an innovative health educational game named Schisto and Ladders™ as a tool for teaching simple schistosomiasis transmission, control, and prevention messages to school aged children. School children in the intervention group had more increase in the knowledge about schistosomiasis than their counterparts in the control group. The significant improvement in the knowledge of school children about the causes of urinary schistosomiasis and transmission after playing Schisto and Ladders™ points to the potential of this game to complement and sustain the gains of school-based mass drug administration programmes. It was evident that captions and pictures used in development and designed of games are essential ingredients for learning [22]. This finding supports the hypothesis that visual games can be helpful in the control of disease as shown by the development of an animated health education cartoon at preventing worm infection in Chinese school children [23, 24]. It is thus imperative to find innovative ways, such as the use of games with health education messages to teach children about diseases, methods of transmission, personal and domestic hygiene and proper health seeking behaviour in endemic schistosomiasis communities. Focus group discussions showed that the pupils liked the game wanting to play the game every day to learn more about schistosomiasis.

Behavioural change indicated by the frequency of visit to the dam water, though reduced, did not markedly change in the intervention group after playing the Schisto and Ladders game. This observation may be due to the absence of alternative water sources in the community, making it difficult if not impossible for the pupils not to visit the dam water despite their awareness of contracting schistosomiasis. Pupils majorly reduced frequency of playing in water, but the same could not be said about other water related house chores activities like washing and bathing which they are likely to be compelled to do by parents and guardians. Provision of alternative safe water supply through repairs and rehabilitation of existing borehole in the community will help in reducing water contact activities of school children with dam water.

Health education among peers has been recognized to play a leading role in catalysing behavioural change in school children. The findings of this study showed that a platform of cross-communication exists among peer groups even within schools. Pupils within the control group did enquire from those within the intervention group what their game was? Although not intended, we found this scenario difficult to control as the nature of the school settings allow pupils to mix freely during short-breaks and closing periods. However, the effect of this was limited by pupils not been aware of post-test assessment and FGDs. Also, game playing was independently handled by different teachers in different arms of the classes. This cross communication among peers can, however, be further utilized in improving the delivery of health education messages in schools and beyond as parents learn easily from their wards, and acquired knowledge is usually transferred from school to home and communities.

The major limitation of this study is the sample size, which reduced the power and limited the conclusion that can be drawn from the study. Also, the choice of control group being in the same school also affected the study as there was evidence of cross-communication between intervention and control group. Further study with larger sample size and in different cultural settings with more schools participating is needed to validate the game.

## Conclusions

This study demonstrated the potential of the health education board game Schisto and Ladders™ for teaching basic health education and promoting behavioural changes among schoolchildren in a friendly and enjoyable way. We believe it can be use in schistosomiasis endemic communities to substantially increase compliance to mass drug administration campaign and reduce reinfection rate among schoolchildren.

## Abbreviations

KAP: knowledge, attitude, and practices
FGD: focus group discussion
